# User Perspectives of a Web-Based Data-Sharing Platform (Open Humans) on Ethical Oversight in Participant-Led Research: Protocol for a Quantitative Study

**DOI:** 10.2196/10939

**Published:** 2018-11-28

**Authors:** Marta Fadda, Anna Jobin, Alessandro Blasimme, Bastian Greshake Tzovaras, Mad Price Ball, Effy Vayena

**Affiliations:** 1 Health Ethics and Policy Lab Department of Health Sciences and Technology ETH Zurich Zurich Switzerland; 2 Open Humans Foundation Boston, MA United States; 3 Division of Environmental Genomics and Systems Biology Lawrence Berkeley National Laboratory Berkeley, CA United States

**Keywords:** ethics, data sharing, patient participation, patient-generated health data, survey, questionnaire, mobile phone

## Abstract

**Background:**

Advances in medicine rely to a great extent on people’s willingness to share their data with researchers. With increasingly widespread use of digital technologies, several Web-based communities have emerged aiming to enable their users to share large amounts of data, some of which can possibly be employed for research purposes by scientists, or to conduct participant-led research (PLR). Scholarship has recently addressed the necessity of interrogating how existing ethical standards can and should be applied and adapted in view of the specificities of such Web-based activities. So far, no study has explored participants’ beliefs about and attitudes toward ethical oversight when it comes to platforms that involve medical data sharing.

**Objective:**

This paper presents the protocol for a survey study aimed at understanding users’ beliefs about Web-based data-sharing platforms regarding how research ethics principles should be applied in such a setting. Furthermore, the study aims at quantitatively assessing the relationship between participants’ perspectives on ethical oversight and other variables such as previous participation in research, beliefs about data sharing, and attitudes toward self-experimentation.

**Methods:**

We are conducting a Web-based survey with users of a popular Web-based data-sharing platform, Open Humans. The survey has been sent to approximately 4640 users registered for the Open Humans newsletter. To fill out the survey, participants need to have an account on Open Humans. We expect a 5%-10% response rate (between 200 and 400 completed surveys out of approximately 4000 survey invitations sent). Independent variables include past data-sharing behavior and intention, beliefs about data sharing, past participation in research, attitudes toward self-experimentation, perceived knowledge of the platform’s guidelines and terms, perceived importance of having transparent guidelines, and governance-related beliefs. The main dependent variable is participants’ expectations regarding who should ensure that ethical requirements are met within research projects conducted on open data-sharing platforms, based on Emanuel et al’s ethical framework. We will use chi-square tests to assess the relationship between participants’ expectations regarding ethical oversight and their past behavior, future intentions, beliefs, attitudes, and knowledge.

**Results:**

Data collection started on June 13, 2018. A reminder to fill out the survey was sent to participants in mid-July. We expect to gain insights on users’ perspectives on the ethical oversight of Web-based data-sharing platforms and on the associated experiences, beliefs, and sociodemographic characteristics.

**Conclusions:**

When digital tools allow people to engage in PLR including medical data, understanding how people interpret and envision the ethical oversight of their data-sharing practices is crucial. This will be the first study to explore users’ perspectives on ethical oversight of Web-based data-sharing platforms. The results will help inform the development of a framework that can be employed for platforms hosting various kinds of research projects to accommodate participants’ ethical oversight needs.

**International Registered Report Identifier (IRRID):**

RR1-10.2196/10939

## Introduction

### Toward Participatory Medicine

Medicine is undergoing a great revolution that is radically transforming health care by not only improving diagnostics and therapeutics but also providing new understandings of disease prevention [1,2]. Contributing to such transformation are some intimately intertwined factors such as the introduction of systems medicine (application of systems biology approaches to disease); big data (availability of large quantities of data); new technologies (that allow study of new magnitudes of individuals’ data); and patients’ increasing participation, engagement, and involvement in health care [1-6]. Large amounts of data are necessary to make sense of systems medicine, and, in turn, sophisticated technologies are crucial for big data to be adequately analyzed and interpreted. Similarly, individuals’ participation, engagement, and involvement in health care are crucial for a constant supply of big data because, by sharing their data with research, individuals can contribute to the accumulation of a vast amount of information [7]. The convergence of these elements is typically referred to as “4P medicine,” a term initially coined by systems biologist Leroy Hood to indicate a type of medicine that is predictive, preventive, personalized, and participatory at the same time [2,4,8-11]. However, 4P faces both technical and societal challenges [2]. While there appear to be rapid technical developments in the capacity to process people’s data, the transition from a reactive to a proactive, empowered approach to medicine seems to pose greater difficulties [1-3]. The key concept of 4P medicine that “the individual is at the center of action-taking related to health and health care” [12] raises the question of not only how to best educate patients, physicians, and public about the opportunities offered by 4P medicine but also how to acquire the amount and type of data necessary for predictive and personalized medicine to be realized [2,12-14]. Reaching out to larger communities of individuals may require looking beyond the traditional researcher-led enrollment system conducted in university hospitals and research centers. Patient-activated networks and patient-initiated research activities may help address this challenge [2,3]. According to many, it is exactly these patient-initiated phenomena that will be the most powerful tools in the strive to push forward the 4P agenda because they may increasingly provide data while fostering individuals’ empowerment and boosting transparency and accountability in science at the same time [3,12,15-18]. We set out to analyze the opportunities and challenges these activities offer and focus on the ethical oversight requirements of such phenomenon.

### Participant-Led Research

The major strategies adopted to promote a participatory approach to medicine involve increasing individuals’ participation, engagement, and involvement in their health and in health research [5,19]. According to Woolley, participation in medical research encompasses activities that involve not only an active, intentional role but also more passive forms of inclusion [5]. The concept of public engagement in scientific studies, on the other hand, does not depend on individuals’ participation in research, that is, individuals can feel engaged even if they do not actively participate in the research. Engagement can be higher or lower according to scientists’ effort to communicate their intentions and request public’s collaboration in collecting data through so-called participant-centric initiatives [5,20]. Finally, involvement characterizes activities where members of public can play an active role in initiating research, selecting the scientific questions to address, and designing a study and implementing it [5,21]. This latter type of initiatives is commonly referred to as participant-led research (PLR), participant-driven research, or participatory research, and it includes a wide spectrum of approaches such as self-experimentation, self-surveillance, analyses of genetic information, and genome-wide associated studies [22,23]. In this study, we decided to adopt the label “PLR” over others because we believe it better emphasizes the main characteristic of this activity, which is not only initiated but also conducted by those who participate in it.

PLR has been facilitated by the integration of a wide range of increasingly affordable technologies into everyday life like computers, smartphones, tablets, and wearable gadgets and by the emergence of social media platforms where people can nowadays share (health-related) information about themselves to be used for a variety of research purposes [23]. For example, platforms like PatientsLikeMe and the Quantified Self offer people the opportunity to design, conduct, and analyze their own studies by uploading different types of data about themselves [12,18,24]. An example of a successful PLR initiative, the results of which were eventually published in an international scientific journal, is the lithium study, in which a group of patients suffering from amyotrophic lateral sclerosis and belonging to the PatientsLikeMe community initiated and conducted a study on the effects of lithium on their condition [25]. The study findings were later confirmed by a standard clinical trial [26]. While the lithium study involved testing a substance on one’s own body, most PLR activities involve uploading one’s data to Web-based platforms (often genetic data derived from direct-to-consumer genetic testing) with the scope of starting or contributing to a research project [15,27,28]. Users may want to publicly share their data on Web-based platforms for a variety of reasons. For example, studies have found that users may want to learn about themselves or contribute to the advancement of medical research and, in the case of genetic and genomic data, want to improve the predictability of genetic testing because they find it fun to explore genotypic and phenotypic data [29,30-34].

PLR activities have some clear limitations, such as an evident self-selection bias due to the fact that its participants usually come from a selected, hyperactivated, and highly educated subset of the general population, leading to little variance in the sociodemographic characteristics of those who join it [35]. However, there is much consensus on the opportunities offered by this novel approach to science [19,22]. First, PLR promotes individuals’ empowerment, one of the pillars of the patient-centered health care model, by allowing people to make more informed and autonomous decisions on their own health [22]. From a patient or participant perspective, taking part in PLR could fulfill individuals’ need for involvement and self-determination that can otherwise be lacking in investigator-led research and that is currently urged in patient care [36]. It can also create opportunities for social support among individuals sharing the same condition or health concerns [21]. PLR supports the democratization of research, a system where anyone has a human right to contribute to science and actively participate in the research process, and significantly helps cut research-related recruitment and logistic costs [18,37,38]. Furthermore, such activities can provide great support to large-scale and longitudinal research studies, accelerate the pace of their execution, and explore areas that standard medical research often overlooks or cannot reach [13,22,39]. Just as in other forms of crowdsourcing, the underlying belief that fuels PLR activities is that the more people are allowed to participate, the more accurate and complete the generated information will be [40]. However, due to the general lack of qualified supervision that distinguishes standard research practice, these bottom-up initiatives challenge existing ethical paradigms and raise questions that scholars have only recently started to address [13,40].

### Ethical Oversight of Participant-Led Research

Just like investigator-led research, PLR poses evident questions related to its ethical, legal, and social implications, potentially resulting in barriers to the optimal integration of its outcomes into scientific evidence and, ultimately, into health care [3]. Beyond issues of accountability, the main concerns are whether PLR can be conducted (1) in a scientifically thorough fashion and (2) in an ethically appropriate manner [14,37]. The first concern follows the consideration that being self-reported, self-collected, and mostly generated without an experienced researcher’s supervision, data produced by PLR may not meet the highest scientific standards characterizing investigator-led research and contain major biases that may lead to questioning data’s reliability and validity [22,26]. In response to this worry, recent scholarship has proposed that PLR can, at least in principle, reach the same level of scientific accuracy of standard research, provided that participants are adequately trained on how to collect and report their data [16,41]. Furthermore, with the widespread use of these approaches, PLR holds the promise of introducing increasingly novel methods for validating its results to secure their publication in international journals and their integration into health care practice [19]. The second concern builds on the assumption that PLR activities bear potential risk of harm for those taking part in them [15,23]. For example, testing off-label drugs without a researcher’s supervision might lead to serious health consequences as much as sharing one’s identifiable genetic data on Web-based platforms may lead to privacy issues that can result in discrimination by employers or insurance companies [7].

Following these considerations, some scholars have argued that PLR should be ethically regulated, and a debate is currently taking place regarding what forms of ethical oversight mechanisms should be adopted in such contexts [16]. While some scholars believe that we should try to capture such research within existing regulatory frameworks (requiring, for example, ethical review by an Institutional Review Board; IRB) [42], Vayena et al have proposed that existing ethical standards should be applied to the specificities of participant-led health research with alternative mechanisms [16]. They have proposed a distinction into 3 categories representing different levels of similarity between a given PLR project and standard research, the level of risk involved, and the type of agent conducting the research; they suggest crowdsourcing review as an alternative method of ethical oversight [12,16,21,23,42]. In particular, a given PLR activity will fall into the first category (and, thus, it will be subject to the standard form of ethics review) if it is performed by state or for-profit institutions; if the activity does not meet the “institution-plus” criterion, it will fall either into the second category (if it involves more than minimal risk to participants) or in the third category (if it involves no more than minimal risk to participants) [16]. While the second category will demand a form of ethics review equivalent to an expedited review (eg, a faster review conducted by the IRB chair and one or more experienced reviewers), no formal ethics review is morally required for the third category [16].

The need to adapt ethical standards to PLR also finds justification in the great amount of evidence showing that participants are more willing to donate their data when an ethical review body has approved the study protocol [43]. But establishing the appropriate ethical oversight mechanism in PLR is not without challenges. In fact, these activities represent a revolutionary movement in contrast to mainstream research, and, as such, they often position themselves as opposed to the traditional elitism of standard research practice [38,44]. Furthermore, applying the same mechanisms of ethical oversight that are also employed to review standard research is likely to impose a burden on PLR in terms of finances, time, and logistics, with the possible consequence of discouraging participation [13,21]. Thus, we can expect some forms of resistance by this novel approach to science against any top-down attempts to regulate it.

### Objective of the Study

The literature suggests a substantial lack of information on individuals’ perspectives regarding ethical oversight in participant-led health research [13,16,38,42]. Previous research has explored the reasons why users decide to publicly share their data on Web-based platforms for research purposes and what prevents them from doing so [29], but no study has so far investigated participants’ perspectives on what ethical oversight methods should be in place in such settings. Even scholarship from other disciplines that has investigated users’ attitudes toward Web-based platform policies has so far addressed issues such as privacy [45] and copyright [46], but overlooked medical research ethics. Considering that most participant-led health research activities take place on Web-based platforms and involve publicly sharing individuals’ data in anonymized, coded, or identifiable forms, the goal of this study is to investigate whether the users and visitors of a Web-based data-sharing platform apply the same ethical principles of standard research to Web-based data sharing and, in particular, to Web-based participant-led health research projects. Furthermore, we aim to investigate the mechanisms of ethical oversight that users think should be adopted in such a context.

## Methods

### Study Design

We will adopt survey methodology in this study. We target the users of the Web-based data-sharing platform Open Humans who have subscribed to the Open Humans newsletter [47]. Initially, we developed a conceptual model that seeks to explain individuals’ ethical oversight expectations of data-sharing platforms with their past data-sharing behavior, their previous participation in research, their perceived importance of having transparent guidelines on Web-based data-sharing platforms and perceived knowledge of them, their attitudes toward data sharing and self-experimentation, and their future data-sharing intention (see Multimedia Appendix 1). Subsequently, we developed a Web-based questionnaire using the survey platform SurveyMonkey [48] on the basis of the literature discussing the main ethical principles applying to standard research and, in particular, Emanuel et al’s ethical framework for clinical research [49]. We developed items to measure the variables that potentially have a relationship with users’ opinions on ethical oversight, such as past data-sharing behavior [29], previous participation in research [50], attitudes toward self-experimentation [51,52] and data sharing [30-34], perceived importance of having transparent guidelines, and knowledge about the platform’s guidelines [30,31]. To content and face validate the questionnaire, we conducted a pretest with a convenience subsample of 6 participants. We contacted potential pretest participants through the Open Humans platform and asked them to fill out the Web-based survey by providing specific feedback on the clarity and appropriateness of each survey item. Data collection continued until data saturation was achieved.

Once the pretest had been conducted and the survey questions refined, we created a research project on a dedicated page of the Open Humans platform where we described the scope of the study and provided a link to the Web-based survey. Open Humans research projects are meant to ask an engaged audience of participants to join and contribute to research. Past and current research projects include the Genevieve Genome Report (matching participants’ genome against public variant data), the Twitter Archive Analyzer (to explore social media usage), and the Keeping Pace project (seeking to study data about how participants move around and to understand how seasons and local environments influence their movement patterns). Altogether, Open Humans research projects have so far involved more than 3000 users.

An invitation to visit the research project Web page and fill out the Web-based survey was sent through the Open Humans regular newsletter to all subscribers (approximately 4640 users) on June 13, 2018. Those who do not have an account on Open Humans are requested to create one to be able to fill out the survey. We expect a response rate of 5%-10% (between 200 and 400 completed surveys out of approximately 4000 survey invitations sent), in line with previous research [53]. To increase the response rate, a second newsletter including a reminder about our research project and the upcoming deadline was sent to potential participants in mid-July 2018.

### Survey Administration

By clicking on the link to the survey, participants are directed to a dedicated page of the Open Humans platform describing the project’s goal (“Data sharing and ethical oversight”) and its academic and nonprofit nature, providing the name and contact details of the principal investigator, and providing the informed consent form as well as a downloadable version of it. Users can only be redirected to the actual survey if they consent to participate in the study by clicking on the corresponding button. If they agree, the participants are directed to the survey on surveymonkey.com, where a short introduction reminds them about the study’s scope and guarantees that no data are extracted from participants’ accounts. An anonymized unique identifier is attributed to each Open Humans platform account to detect multiple entries from the same individual. If such multiple entries are found from the same individual, we will keep the first entry for analysis. To start the survey, participants are asked to confirm that they “have joined the project on openhumans.org, read the description, and accepted the corresponding consent form.” The survey displays 1-3 questions on each page according to the questions’ length, and participants can review and change their answers through a “back” button. We ensured that survey questions are appropriately displayed on mobile phones. Completion of the survey is estimated to take approximately 15-18 minutes. To avoid missing data, answers to the questions are mandatory, except for sociodemographic variables. We offer no remuneration for participation in this study. However, we ask participants whether they would like to receive their responses to the survey and the aggregated answers from all respondents for comparison.

### Analysis

Once the survey has been closed, we will import the data into SPSS (IBM Corp, version 24.0). We will compute frequencies and correlations and use chi-square tests to assess the relationships between participants’ ethical oversight expectations and their past behavior, future intentions, beliefs, attitudes, and knowledge. A priori power analysis suggests that a sample size of 142 respondents would be adequate to detect a moderate effect with alpha=.05 and power=.8 [54]. Thus, our proposed minimum sample size of N=200 will be more than adequate for the main objective of this study. Data missing at random will be handled using multiple imputation [55]. Depending on the nature of the comments provided in the open-ended questions, we might also be able to gather important qualitative insights. In this case, two researchers will code the comments and label them. Similar labels will then be merged into broader themes to provide a comprehensive description of the findings [56].

### Institutional Review Board Approval

The study protocol, including survey questions, has been approved by the Ethics Commission of the Federal Institute of Technology, Zurich on May 7, 2018, with the title “Ethical oversight in online data-sharing platforms” (Ref.: EK 2018-N-36). We expect there will be no risks to participants in this study.

### Measures

This is the first study to explore individuals’ ethical oversight expectations regarding research projects on open data-sharing platforms. For this reason, we developed our own conceptual model in an effort to explain participants’ ethical oversight expectations through their past data-sharing behavior, their previous participation in research, their perceived importance of having transparent guidelines on Web-based data-sharing platforms and perceived knowledge of them, their attitudes toward data sharing and self-experimentation, and their future data-sharing intention (see Multimedia Appendix 1). To build measures for our variables of interest, we relied on the literature on the ethical principles of standard research [49,57-59], on what characterizes PLR [12,16,19,37,60-62], and on barriers to and facilitators of data sharing in varous contexts (eg, biobanking) [30-32]. Below we have described how survey questions were created on the basis of the literature across all variables. The survey questions can be found in Multimedia Appendix 2.

### Independent Variables

#### Past Data-Sharing Behavior

The level of users’ engagement with data sharing represents a key variable for comparison between the ethical oversight expectations of more or less engaged users. We speculate that the amount of data users have publicly shared in the past will be significantly linked to the type of ethical oversight mechanism preferred. We will measure past data-sharing behavior with 4 questions. A filter question will ask participants whether they have ever tracked, collected, or been in possession of different types of data (such as vital signs, stress levels, and mood). The next 2 questions will target specific types of data selected by participants and will ask participants whether they have ever shared that data on the Open Humans platform and on any Web-based platforms other than Open Humans. We extracted the list of types of data from the 2014 Report of the Health Data Exploration Project and adapted it to the digital context [63]. The fourth question will focus on genetic data and will ask participants whether they have ever shared this type of data in any of the most popular genetic data-sharing platforms (eg, OpenSNP, SNPedia, and DNAland). A multiple answer option will be provided for all questions (see Multimedia Appendix 2).

#### Intention to Share Data in the Future

Intention is a well-known antecedent of actual behavior [64]. We will measure participants’ intention to share their data with a matrix question asking to what extent participants would agree to share their data for research purposes if they were asked to do so in the future. The list of types of data will be the same employed to measure past data-sharing behavior extracted from the 2014 Report of the Health Data Exploration Project and adapted to the digital context [63], and we will collect answers on a 5-point Likert scale measuring agreement and anchoring at “strongly disagree” and “strongly agree.”

#### Beliefs About Data Sharing

Data-sharing beliefs have been found to be linked to intention to share and interest in sharing [30-33]. To measure participants’ beliefs regarding data sharing, we will employ 5 items adapted from a previous survey study involving users publicly sharing their data, for instance, “sharing my data makes me feel part of scientific research,” “I want to contribute to the advancement of medical research,” and “I want to compare my data to that of other people” [29]. Answers will be collected on a 5-point Likert scale measuring agreement and anchoring at “strongly disagree” and “strongly agree.”

#### Past Participation in Research

Previous research has found that 15.57% of surveyed individuals who had publicly shared their data were or had been research participants [29]. To measure participants’ previous participation in research, we will ask them whether they have ever taken part in a clinical trial, a survey or questionnaire study, a qualitative study (eg, interview or focus group), a nonclinical trial, or another type of research study they might want to specify. Answer options will include (1) “no, never”; (2) “yes, in the past week”; (3) “yes in the past month”; (4) “yes, in the past 6 months”; (5) “yes, in the past year”; and (6) “yes, more than 1 year ago.”

#### Attitude Toward Self-Experimentation

Self-experimentation is one of the forms that PLR activities can take [52,65]. However, self-experimentation can, in turn, take different shapes, according to the level of risk involved in the experimental activity [51,52]. Due to the lack of studies on people’s attitudes toward self-experimentation, we speculate that individuals with a positive attitude toward the most extreme forms will be significantly more likely to expect less institutional ethical oversight mechanisms. To measure participants’ attitudes toward self-experimentation, we will provide 3 brief scenarios describing different examples of self-experimentation, from low-risk to high-risk behaviors [52,65]. Answers will be collected on a 5-point Likert scale measuring approval and anchoring at “strongly disapprove” and “strongly approve”; Multimedia Appendix 2 provides a full description of the scenarios.

#### Perceived Knowledge of the Guidelines and Terms of Open Humans

Perceived knowledge about what is involved in an action or a decision is a known predictor of individuals’ self-determination in many behavioral and decisional contexts [66]. Individuals’ self-determination might, in turn, regulate the expectation of a more or less rigorous institutional review for a given PLR project. We speculate that participants with higher levels of perceived knowledge regarding the platform’s guidelines will be significantly more likely to indicate less institutional ethical oversight mechanisms for public data-sharing activities because they feel more autonomous in their decision making. This variable will measure the perceived knowledge of the main guidelines and terms of use of the Open Humans platform, such as privacy, projects, and community guidelines. Participants will be asked to indicate how familiar they are with each guideline. We purposely decided not to include objective questions (such as a quiz) because we do not want our participants to feel they are being tested. However, to detect automatic replies and those conforming to social desirability, we have added control questions about hypothetical guidelines that do not actually exist, and we will, thus, be able to filter answers of the individuals who state they are “(extremely) familiar” with these. Answers will be measured on a 5-point Likert scale anchoring at “not familiar at all” and “extremely familiar.” This question is meant to measure, in general, the extent to which participants perceive themselves knowledgeable about the platform’s guidelines. We employed broad, recognizable labels so that participants can more easily assess whether they are in possession of that information regarding the platform’s guidelines. Perhaps then, our labels do not exactly reflect the names appearing on the website. We will calculate a summative score for this set of questions, excluding the control questions.

#### Perceived Importance of Having Transparent Guidelines and Other Governance-Related Variables

Barazzetti et al have addressed the expectations of people in regulatory governance of biobank research. Although their study was not about a Web-based platform, their results underline the need to inquire further into the alignment between perception, regulation, and actual communication [67]. Additionally, Earp et al looked closely at users’ expectations of information regarding privacy and compared them with both actual regulation and the existing policy statements, finding important divergences [68]. Fiesler et al focused on users’ expectations compared with actual legislation regarding copyright aspects, but their article also gives a useful overview of previous work related to the perception of Terms of Services more generally [69]. In an effort to assess users’ governance-related expectations, we decided to measure the extent to which participants believe it important to have transparent guidelines on both the Open Humans platform and other open data-sharing platforms. Main guidelines will include areas such as privacy, projects, and community. Answers will be measured on a 5-point Likert scale measuring importance and anchoring at “not important at all” and “extremely important.” The survey will also include a variety of questions aimed at measuring participants’ perceptions of other aspects of governance of the Open Humans platform such as (1) the perceived influence of the nonprofit status of Open Humans on the decision to sign up or navigate the platform (measured on a 5-point Likert scale anchoring at “not influential at all” and “extremely influential”); (2) beliefs regarding who should make decisions about the Open Humans platform (eg, users taking part in research projects, any users of the platform, and independent nonqualified ethical committee); (3) participants’ desire to be involved in decisions about the governance of the Open Humans platform (measured on a 5-point Likert scale anchoring at “strongly disagree” and “strongly agree”); and (4) amount of time participants are willing to invest into governing the Open Humans platform (measured on a 5-point Likert scale anchoring at “none at all” and “a great deal”).

#### Dependent Variable

##### Expectations Regarding Ethical Oversight

Participants’ expectations regarding ethical oversight mechanisms on Web-based data-sharing platforms will be measured with 11 question asking them who they think should ensure that 6 ethical requirements for clinical research [49] are met within the research projects conducted on Web-based data-sharing platforms. The ethical framework that guided the creation of these questions is Emanuel et al’s “7 ethical requirements” framework [49]. Each ethical requirement will be covered by one or more questions. We decided to not include 1 of the 7 ethical requirements, that is, “value,” because it has to do with the dissemination of the research results and the potential of the research to increase knowledge [49]. This requirement dictates whether the study will receive funding and, thus, represents a preliminary evaluation of the research project that does not match the core characteristic of PLR activities, the peculiarity of which is a noninstitutional, bottom-up approach [16]. The ethical requirements selected by us are (1) scientific validity (“Who should ensure that the research is conducted in a methodologically rigorous manner?”); (2) fair subject selection (“Who should ensure that recruitment is fair and balanced and not restricted to certain populations on the basis of convenience or efficiency or by exploiting vulnerable individuals or communities?”); (3) favorable risk-benefit ratio (eg, “Who should ensure that potential risks to individual subjects are minimized?”); (4) independent review (eg, “Who should ensure a research project’s compliance with ethical requirements?”); (5) informed consent (eg, “Who should ensure that individuals are accurately informed about the purpose, methods, risks, benefits, and alternatives to the research?”); and (6) respect for potential or enrolled subjects (“Who should ensure that individuals’ privacy is respected by managing the information in accordance with confidentiality rules?”). Participants will be asked to choose among 9 answer options, namely (1) “No one in particular, because it does not apply”; (2) “No one in particular, for another reason (please specify)”; (3) “Users participating in the project”; (4) “Any registered users of the platform”; (5) “The creators or directors of the project”; (6) “The creators or owners or directors of the platform”; (7) “An independent, nonspecialized committee (eg, a group of citizen volunteers)”; (8) “An independent ethics committee (eg, a university IRB)”; and (9) “Other” (with the possibility of entering text).

##### Sociodemographic Variables

We will also ask participants a number of questions pertaining to their sociodemographic status, such as perceived health status, presence of a chronic condition (both referring to the participant and to the participant’s family members), gender, age, ethnicity, country of residence, education, marital status, number of children, employment status, health and life insurance coverage, and past experience in the health field.

## Results

We initially conducted a pretest with a convenience sample of 6 users of the Open Humans platform; subsequently, we refined the survey questions and started data collection on June 13, 2018. Results will provide information on not only which mechanisms of ethical oversight participants expect to be implemented within research projects conducted on the Open Humans platform but also the behavioral and psychosocial features of the Open Humans users participating in the study. Although we expect mainly US participants to join the study (as Open Humans is based in the United States), users from other countries can also participate. In case we detect important differences based on national contexts, we will take into account the main US and European laws and regulations on privacy that are important for ethical considerations for the analysis and interpretation of responses.

We speculate that participants will envision stronger ethical oversight mechanisms for principles like autonomy, while they will perceive other principles such as privacy and confidentiality as less applicable to this context. This is because open data sharing is a voluntary activity that involves participants’ affirmation of their autonomy, conducted without any privacy protection (data can be accessed by anyone) [16]. Furthermore, we hypothesize that previous participation in research, past data-sharing behavior, perceived importance and knowledge of Open Humans guidelines and terms, attitudes toward self-experimentation and data sharing, and future data-sharing intention will have significant relationships with their expectations regarding ethical oversight. In particular, we formulated the following hypotheses:

H_1_: Participants will highly value certain principles such as respect for autonomy, while they will deem other principles such as privacy and confidentiality less important in this setting. Due to the public nature of open data sharing (no privacy protection is offered), participants might assume that they have relinquished their privacy, accepting that it cannot be protected. On the other hand, those publicly sharing their own data are likely to expect that their self-determination is respected and enhanced.

H_2_: Participants with previous participation in research will be significantly more likely to indicate that an independent ethics committee should be in charge of applying the main ethical principles on Web-based data-sharing platforms compared with those with no previous participation in research. Because of their previous exposure to the investigator-led research model, they might overgeneralize and assume that standard ethical review should also apply to PLR [43].

H_3_: Participants with more positive attitudes toward self-experimentation will be significantly more likely to indicate that users participating in the project should themselves be in charge of applying the main ethical principles on Web-based data-sharing platforms. Self-experimentation represents an expression of self-determination or autonomy that can regulate the expectation regarding the type of ethical oversight applying to PLR [51,52,65].

H_4_: Participants with lower perceived knowledge of the platform’s guidelines will be more likely to indicate that an external review board should be in charge of applying the main ethical principles on Web-based data-sharing platforms. Multiple studies based on self-determination theory have found perceived knowledge to be significantly and positively correlated with self-determination [66], which might regulate one’s ethical oversight expectation (more or less institutional).

## Discussion

### Study Rationale

Research is likely to benefit from fostering more engaged research participation [3]. The integration of digital tools into everyday life is facilitating people’s active involvement in research, and PLR activities are becoming an increasingly popular phenomenon [70]. PLR initiatives hold great promise for science, not only because they are a tangible expression of a response to the push for a more participatory medicine but also because they offer significant opportunities for advances in a variety of fields by offering novel solutions to complicated challenges [22]. Yet, if not duly overcome, some challenges are likely to become burdensome bottlenecks to the successful realization of these activities. In particular, ethical oversight mechanisms need to be adapted to PLR to ensure that the same ethical standards of investigator-led research are fulfilled while PLR values are, at the same time, respected and promoted [16,21,23]. Some alternative solutions to standard ethics review (such as crowdsourcing ethics review) have been proposed [16], but evidence is currently missing on the perspectives of individuals who are directly involved in PLR activities. Our study represents the first attempt to explore what ethical oversight mechanisms users and visitors of a Web-based platform hosting research projects think should be in place. The results will also inform a broader debate on PLR and its potential impact outside the medical realm. In fact, the potential significance of PLR is much broader than medical research and also invests health or wellness research and nonmedical human research (this reflection was contributed by an anonymous reviewer). On the basis of the type of data that our participants report to be engaged with, we will be able to establish which fields of application are suitable for the preferred PLR ethical oversight mechanisms.

### Limitations

A number of limitations to this study are worth mentioning. First, we will recruit our participants from a relatively small, US-based data-sharing platform; therefore, the results—while representative of the Open Humans population—might not be generalizable to other platforms. The decision to restrict our inquiry to the Open Humans community has been dictated by the availability of the platform to join this study. Furthermore, because this is the first study of this kind, we aim to collect an initial set of information from a single, circumscribed group of people. A second limitation is that while the users of Open Humans can share their data for research purposes by contributing to dedicated research projects, other platforms such as PatientLikeMe represent more common venues to start leading PLR activities. However, Open Humans represents a fast-growing platform for initiating and conducting research projects with a diverse and highly involved community. Third, we will ask our participants what potential ethical oversight mechanisms they think should be in place within research projects on the Open Humans platform, thus, introducing a hypothetical bias [71]. Soliciting their opinions solely on already established oversight mechanisms might have provided a more valid and reliable account, but in the current state, it remains unclear what these would have entailed. Finally, we will compare engaged and nonengaged users on the basis of their answers to the past data-sharing behavior questions. Having access to their account activity data would have represented a more reliable and objective measure of engagement. However, we purposely decided not to include this type of data to ensure that participants feel comfortable in sharing their opinions and experiences with the research team.

### Future Research

This study is the first attempt to elicit individuals’ perspectives on the ethical oversight of Web-based PLR activities and to compare such views with their experiences, beliefs, knowledge, and sociodemographic characteristics. Future research should replicate our effort in novel methodological and contextual ways. First, because our study will follow a quantitative approach, qualitative research might be a valuable approach to further investigate people’s expectations regarding which forms of ethical oversight should be applied to PLR. Second, as we restricted our enquiry to a single platform, exploring and comparing different geographical settings will certainly provide more insights on users’ expectations and allow for a better refinement of any policy recommendations. Furthermore, it would be interesting to study whether practitioners’ beliefs about ethical oversight can differ on the basis of research type (including medical research, wellness research, or scientific research involving human data).

To maximize the extent to which it can benefit from research, society has a legitimate priority and concern about protecting research participants and ensuring that a high-quality research is conducted in an ethical manner [23]. As PLR is capable of producing generalizable scientific knowledge, just as standard research, it is crucial to understand what criteria are important to PLR participants to determine who should be in charge of ensuring that standard ethical principles are satisfied [23].

## Appendix

Multimedia Appendix 1 Conceptual model.

Multimedia Appendix 2 Survey.

## Abbreviations

4P medicine: predictive, preventive, personalized, and participatory medicine
IRB: Institutional Review Board
PLR: participant-led research
