# Supplementary figures and images for: User Perspectives of a Web-Based Data-Sharing Platform (Open Humans) on Ethical Oversight in Participant-Led Research: Protocol for a Quantitative Study
Source: JMIR Res Protoc. 2018 Nov 28;7(11):e10939. doi: 10.2196/10939 (PMC6291678; doi:10.2196/10939)

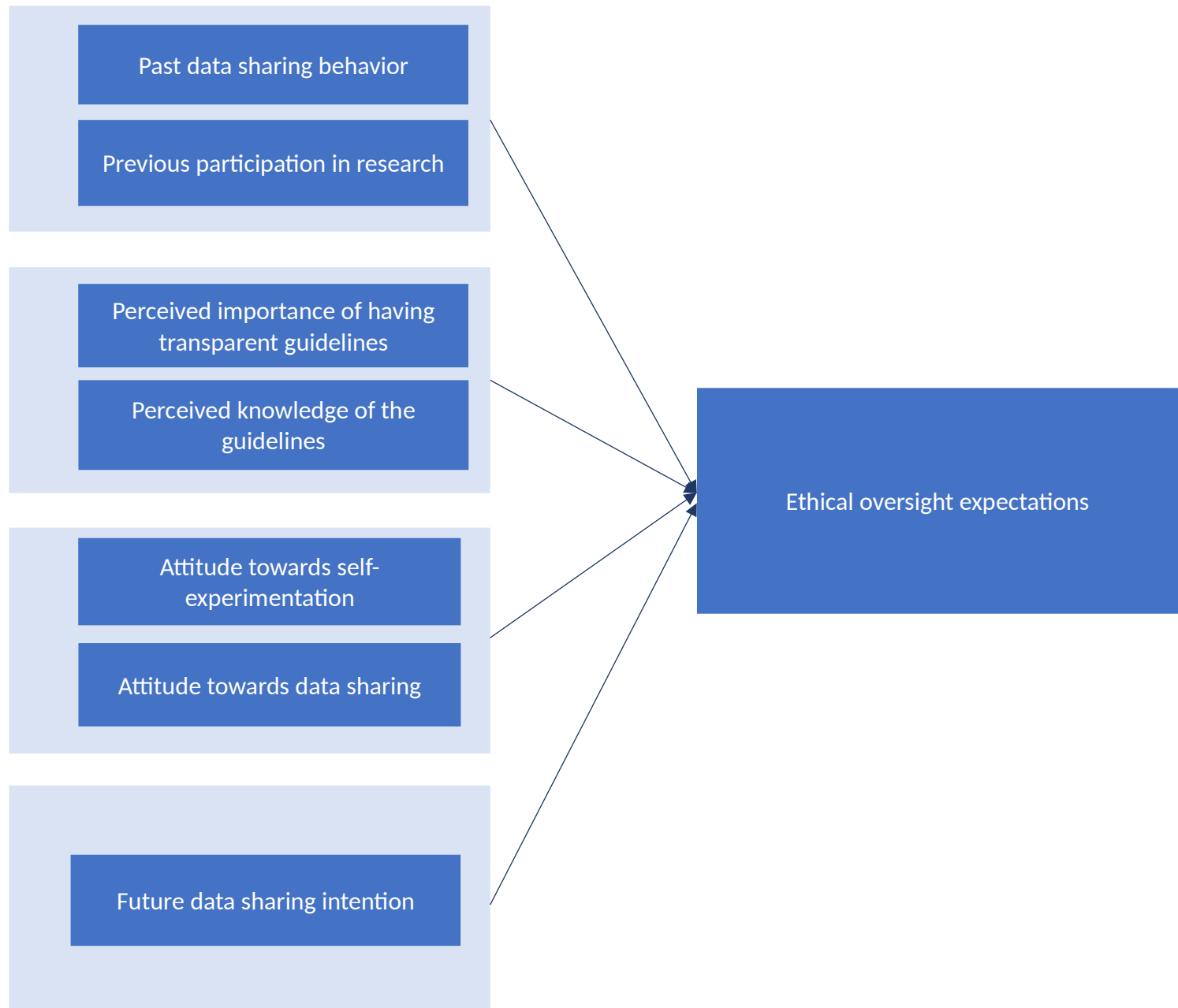

Supplement: Multimedia Appendix 1 [file resprot_v7i11e10939_app1.pdf]
