# Supplementary material for: User Perspectives of a Web-Based Data-Sharing Platform (Open Humans) on Ethical Oversight in Participant-Led Research: Protocol for a Quantitative Study
Source: JMIR Res Protoc. 2018 Nov 28;7(11):e10939. doi: 10.2196/10939 (PMC6291678; doi:10.2196/10939)
Supplement: Multimedia Appendix 2 [file resprot_v7i11e10939_app2.pdf]

Open Humans Survey

Dear participant,

Thank you for taking the time to take part in this survey.

You should have arrived on this page because you joined our project on Open Humans and clicked on “I AGREE” on the consent form. *(If you take this survey without joining the project on Open Humans, your answers will not be counted, even if you click "yes" below.)*

Please note that we have no access to your personal Open Human data and individual answers to the survey will be kept confidential. Therefore, we kindly ask you to answer all questions truthfully. When we ask for your opinions and beliefs, there are no right or wrong answers.

Completion of the survey should take maximum 15 minutes. At the end, we will ask you whether you want to receive your own answers and/or all answers from this project as an aggregate once the study is over. This way, you can compare your own answers with those of the other participants.

The Open Humans project page 'Data sharing and ethical oversight' can be found here.

Governance of Open Humans
*Please tell us your **opinion***

Q1 How familiar are you with how decisions about the Open Humans website (platform) are being made?

Not familiar at all
Moderately familiar
Very familiar

Q2 How familiar are you with the following guidelines and terms of Open Humans?

	Not familiar at all	Moderately familiar	Very familiar
Privacy guidelines			
Project guidelines			
Public data guidelines			
Private data guidelines			
Community guidelines			
Sharing guidelines			
Naming guidelines			
Research guidelines			
Terms of use			

Q3 How important do you find it that <u>Open Humans</u> has transparent guidelines in the following areas?

	Not important at all	Slightly important	Moderately important	Very important	Extremely important
Privacy					
Projects					
Public data					
Private data					
Community					
Sharing					
Naming					
Research					
Terms of use					

Q4 How important do you find it that online platforms in general have transparent guidelines in the following areas?

	Not important at all	Slightly important	Moderately important	Very important	Extremely important
Privacy					
Projects					
Public data					
Private data					
Community					
Sharing					
Naming					
Research					
Terms of use					

Data sharing

*Please tell us about your **data***

Q5 Please select if you have ever tracked, collected, or otherwise been in possession of your own data about:

Genes and/or other genetic data
Vital Signs
Stress Levels
Mood
Physical Activity
Weight/BMI
Diet
Medication
Sleep Patterns
Smoking Patterns
Alcohol Consumption
Environmental Exposure
Meditation
Personal Genetic Test
Menstrual Cycles
Microbiome
Geolocation
Infectious Disease(s) Contracted
Blood Count
Cholesterol Levels
Diabetes
Other Blood Test Results not listed here (please specify below)
Other Chronic Condition or Disease not listed here (please specify below)
Other (please specify)

Data sharing
Please tell us about your ***past practices***

Q6 Please select if you have ever shared on Open Humans your data about:

- Genes and/or other genetic data
- Vital Signs
- Stress Levels
- Mood
- Physical Activity
- Weight/BMI
- Diet
- Medication
- Sleep Patterns
- Smoking Patterns
- Alcohol Consumption
- Environmental Exposure
- Meditation
- Personal Genetic Test
- Menstrual Cycles
- Microbiome
- Geolocation
- Infectious Disease(s) Contracted
- Blood Count
- Cholesterol Levels
- Diabetes
- Other Blood Test Results not listed here (please specify below)
- Other Chronic Condition or Disease not listed here (please specify below)
- Other (please specify)

Data sharing
*Please tell us about your **past practices***

Q7 Please select if you have ever shared on any online platforms other than Open Humans your data about:

Genes and/or other genetic data
Vital Signs
Stress Levels
Mood
Physical Activity
Weight/BMI
Diet
Medication
Sleep Patterns
Smoking Patterns
Alcohol Consumption
Environmental Exposure
Meditation
Personal Genetic Test
Menstrual Cycles
Microbiome
Geolocation
Infectious Disease(s) Contracted
Blood Count
Cholesterol Levels
Diabetes
Other Blood Test Results not listed here (please specify below)
Other Chronic Condition or Disease not listed here (please specify below)
Other (please specify)

Data sharing

*Please tell us about your **past practices***

Q8 Please select if you have ever shared your genetic data on any of the following platforms:

OpenSNP
SNPedia
DNAland
23andMe
AncestryDNA
Heterogeneous
GedMatch
Harvard Personal Genome Project
UK Personal Genome Project
Other (please specify)

Data sharing
Please tell us about a ***hypothetical decision***

Q9 If you were in possession of these kinds of data, which ones would you agree to share for research purposes in the future?

	Strongly disagree (I would never share this data)	Somewhat disagree (I would rather not share this data)	Neither agree nor disagree (It depends, I don't know if I would share this data)	Somewhat agree (I would probably share this data)	Strongly agree (I would absolutely share this data)
Genes and/or other genetic data					
Vital Signs					
Stress Levels					
Mood					
Physical Activity					
Weight/BMI					
Diet					
Medication					
Sleep Patterns					
Smoking Patterns					
Alcohol Consumption					
Environmental Exposure					
Meditation					
Personal Genetic Test					
Menstrual Cycles					
Microbiome					
Geolocation					
Infectious Disease(s) Contracted					
Blood Count					
Cholesterol Levels					
Diabetes					
Other Blood Test Results not listed here					

Other Chronic Condition or Disease not listed here

Comments:

Governance of ethical requirements
*Please tell us your **opinion***

For research projects on online data sharing platforms:

Q10 Who should ensure that the research is conducted in a *methodologically rigorous* manner?
Multiple answers are possible

- No one in particular, because it does not apply
- No one in particular, for another reason (please specify below)
- The users participating in the project
- Any registered users of the platform
- The creators/directors of the project
- The creators/owners/directors of the platform
- An independent non-specialized committee (e.g. a group of voluntary citizens)
- An independent ethics committee (e.g. a University Institutional Review Board)
- Others (please specify below)

Comments:

For research projects on online data sharing platforms:

Q11 Who should ensure that *recruitment is fair and balanced* (and not restricted to certain populations on the basis of convenience, efficiency, or by exploiting vulnerable individuals or communities)?
Multiple answers are possible

- No one in particular, because it does not apply
- No one in particular, for another reason (please specify below)
- The users participating in the project
- Any registered users of the platform
- The creators/directors of the project
- The creators/owners/directors of the platform
- An independent non-specialized committee (e.g. a group of voluntary citizens)
- An independent ethics committee (e.g. a University Institutional Review Board)
- Others (please specify below)

Comments:

For research projects on online data sharing platforms:

Q12 Who should ensure that *potential risks* for individual subjects are *minimized*?
Multiple answers are possible

No one in particular, because it does not apply
No one in particular, for another reason (please specify below)
The users participating in the project
Any registered users of the platform
The creators/directors of the project
The creators/owners/directors of the platform
An independent non-specialized committee (e.g. a group of voluntary citizens)
An independent ethics committee (e.g. a University Institutional Review Board)
Others (please specify below)

Comments:

For research projects on online data sharing platforms:

Q13 Who should ensure that the *potential benefits* for individual subjects are *enhanced*?
Multiple answers are possible

No one in particular, because it does not apply
No one in particular, for another reason (please specify below)
The users participating in the project
Any registered users of the platform
The creators/directors of the project
The creators/owners/directors of the platform
An independent non-specialized committee (e.g. a group of voluntary citizens)
An independent ethics committee (e.g. a University Institutional Review Board)
Others (please specify below)

Comments:

For research projects on online data sharing platforms:

Q14 Who should ensure that the potential benefits for individual subjects or for society are *proportionate* to the risks (or outweigh them)?
Multiple answers are possible

No one in particular, because it does not apply
No one in particular, for another reason (please specify below)
The users participating in the project
Any registered users of the platform
The creators/directors of the project
The creators/owners/directors of the platform
An independent non-specialized committee (e.g. a group of voluntary citizens)
An independent ethics committee (e.g. a University Institutional Review Board)
Others (please specify below)

Comments:

For research projects on online data sharing platforms:

Q15 Who should ensure a research project's *compliance with ethical requirements*?
Multiple answers are possible

No one in particular, because it does not apply
No one in particular, for another reason (please specify below)
The users participating in the project
Any registered users of the platform
The creators/directors of the project
The creators/owners/directors of the platform
An independent non-specialized committee (e.g. a group of voluntary citizens)
An independent ethics committee (e.g. a University Institutional Review Board)
Others (please specify below)

Comments:

For research projects on online data sharing platforms:

Q16 Who should ensure that the *impact of potential conflicts of interest is minimized*?
Multiple answers are possible

- No one in particular, because it does not apply
- No one in particular, for another reason (please specify below)
- The users participating in the project
- Any registered users of the platform
- The creators/directors of the project
- The creators/owners/directors of the platform
- An independent non-specialized committee (e.g. a group of voluntary citizens)
- An independent ethics committee (e.g. a University Institutional Review Board)
- Others (please specify below)

Comments:

For research projects on online data sharing platforms:

Q17 Who should ensure that *individuals are accurately informed* of the purpose, methods risks, benefits, and alternatives to the research?
Multiple answers are possible

- No one in particular, because it does not apply
- No one in particular, for another reason (please specify below)
- The users participating in the project
- Any registered users of the platform
- The creators/directors of the project
- The creators/owners/directors of the platform
- An independent non-specialized committee (e.g. a group of voluntary citizens)
- An independent ethics committee (e.g. a University Institutional Review Board)
- Others (please specify below)

Comments:

For research projects on online data sharing platforms:

Q18 Who should ensure that individuals *understand* this information and its bearing on their own situation?
Multiple answers are possible

- No one in particular, because it does not apply
- No one in particular, for another reason (please specify below)
- The users participating in the project
- Any registered users of the platform
- The creators/directors of the project
- The creators/owners/directors of the platform
- An independent non-specialized committee (e.g. a group of voluntary citizens)
- An independent ethics committee (e.g. a University Institutional Review Board)
- Others (please specify below)

Comments:

For research projects on online data sharing platforms:

Q19 Who should ensure that individuals make a *voluntary and uncoerced decision* whether to participate?
Multiple answers are possible

- No one in particular, because it does not apply
- No one in particular, for another reason (please specify below)
- The users participating in the project
- Any registered users of the platform
- The creators/directors of the project
- The creators/owners/directors of the platform
- An independent non-specialized committee (e.g. a group of voluntary citizens)
- An independent ethics committee (e.g. a University Institutional Review Board)
- Others (please specify below)

Comments:

For research projects on online data sharing platforms:

Q20 Who should ensure that individuals' *privacy is respected* by managing the information in accordance with confidentiality rules?
Multiple answers are possible

No one in particular, because it does not apply
No one in particular, for another reason (please specify below)
The users participating in the project
Any registered users of the platform
The creators/directors of the project
The creators/owners/directors of the platform
An independent non-specialized committee (e.g. a group of voluntary citizens)
An independent ethics committee (e.g. a University Institutional Review Board)
Others (please specify below)

Comments:

Self-experimentation
Below, we present three different cases of self-experimentation
*Please tell us your **opinion***

Scenario 1
A person wants to be more productive in the workplace and, for this purpose, downloads a mobile app to keep track of activities during the workday: time spent on certain tasks, meals and drinks, and interaction with other people. Then the person compares the data across time to understand what affects performance at work.

Q21 How much do you approve or disapprove of the self-experimentation in this example?

Strongly approve
Approve
Neither approve nor disapprove
Disapprove
Strongly disapprove

Scenario 2
A person has a condition that is not properly responding to the treatment prescribed by the clinician. This person begins self-medicating with drugs synthesized at home.

Q22 How much do you approve or disapprove of the self-experimentation in this example?

Strongly approve
Approve
Neither approve nor disapprove
Disapprove
Strongly disapprove

Scenario 3
A person has been overweight for many years despite trying different diets recommended by the clinician. The person decides to change the daily diet and switch from omnivore to vegetarian.

Q23 How much do you approve or disapprove of the self-experimentation in this example?

Strongly approve
Approve
Neither approve nor disapprove
Disapprove
Strongly disapprove

Governance of Open Humans
*Please tell us your **opinion and motivation***

Q24 Who do you think should be able to participate in decisions about the Open Humans platform?

The users participating in research projects
Any registered users of the platform
An independent non-specialized committee (e.g. a group of voluntary citizens)
An independent qualified ethics committee (e.g. a University Institutional Review Board)
The creators/owners/directors of the platform
Others (please specify)

Q25 How important has the non-profit status of Open Humans been on your decision to sign up/navigate the platform?
Not important at all
Not so important
Somewhat important
Very important
Extremely important

Q26 If it were possible, would you personally like to be involved in decisions about the governance of the Open Humans platform?
No, not at all
No, probably not
Maybe, I don't know
Yes, probably
Yes, absolutely

Q27 How much time are you willing to put into governing the Open Humans platform?
No time at all
Only little time occasionally
A few hours monthly
A few hours weekly
As much time as it takes

Q28 Do you have any comments about the governance of Open Humans? Any questions? Any suggestions? Any complaints?

Please tell us about ***yourself***

Q29 To what extent do you agree with the following statements?

	Strongly disagree	Somewhat disagree	Neither agree nor disagree	Somewhat agree	Strongly agree
Sharing my data makes me feel part of scientific research					
I use Open Humans because I want to learn more about myself					
It is fun to explore health data					
I want to contribute to the advancement of medical research					
I want to compare my data to that of other people					

Q30 Have you ever participated in one of the following types of research project?

	No, never	Yes, in the past week	Yes, in the past month	Yes, in the past six months	Yes, in the past year	Yes, more than one year ago
Clinical trial						
Non-clinical trial						
Survey/questionnaire (the current survey notwithstanding)						
Qualitative study (e.g.: interview or focus group)						
Other						

Comments:

Q31 What portion of your medical costs does your health insurance cover?

It covers **all** of my medical costs
It covers **most** of my medical costs
It covers **some** of my medical costs, enough for me not to worry
It covers **some** of my medical costs, but significantly less than needed
It covers **almost none** of my medical costs
I have health insurance but **don't know** how much of my medical costs are covered
I do not have health insurance
Other (please specify)

Q32 Do you have life insurance?

Yes
No
I don't know
I prefer not to answer

Q33 How would you rate the quality of your health?

Poor
Fair
Good
Very good
Excellent
I prefer not to answer

Q34 Do you have any work or study experience in the health field?

Yes
No
Comments:

Q35 Which statement best describes your current employment status?

Working (paid employee)
Working (self-employed)
Not working (temporary layoff from a job)
Not working (retired)
Not working (disabled)
I prefer not to answer
Other (please specify)

Q36 What is the highest level of school you have completed or the highest degree you have received?

Less than high school degree
High school graduate (high school diploma or equivalent including GED)
Some college but no degree
Associate degree in college (2-year)
Bachelor's degree in college (4-year)
Master's degree
Doctoral degree
Professional degree (JD, MD)
I prefer not to answer
Other (please specify)

Q37 Do you suffer from any chronic conditions (e.g.: asthma, diabetes, multiple sclerosis)?

Yes
No
I don't know
I prefer not to answer

Comments:

Q38 Do any of your family members suffer from any chronic conditions (e.g.: asthma, diabetes, multiple sclerosis)?

Yes
No
I don't know
I prefer not to answer

Comments:

Q39 What is your gender?

- Male
- Female
- Non-binary
- Other (please specify)

Q40 What is your age?
18-24
25-34
35-44
45-54
55-64
65+

Q41 How many children do you have?
I don't have children
1
2
3 or more

Q42 What is your marital status?
Married
Widowed
Divorced
Separated
Single
In a relationship
Other

Q43 In which country do you live?

▼ Afghanistan ... Zimbabwe

Q44 If applicable, please select one or more ethnic groups that you consider yourself to be:
White
Black or African American
Hispanic or Latinx
American Indian or Alaska Native
Asian
Native Hawaiian or Pacific Islander
I prefer not to answer
Other (please specify)

That's it, you're done. Thank you very much for your participation.

Would you like to receive your own answers to this survey and/or other participants’ answers as an aggregate?

(If you select "yes" the data will be available in your Open Human account once the study is over. Don't forget to click "DONE".)

Yes, please send me both
Yes, please send me my own answers only
Yes, please send me other participants’ answers (aggregate)
No, thanks
